# Newton shows the light: a commentary on Newton (1672) ‘A letter … containing his new theory about light and colours…’

**DOI:** 10.1098/rsta.2014.0213

**Published:** 2015-04-13

**Authors:** Patricia Fara

**Affiliations:** History & Philosophy of Science Department, Clare College, Cambridge CB2 1TL, UK

**Keywords:** Newton, optics, Descartes, wave theory, refraction

## Abstract

Isaac Newton's reputation was initially established by his 1672 paper on the refraction of light through a prism; this is now seen as a ground-breaking account and the foundation of modern optics. In it, he claimed to refute Cartesian ideas of light modification by definitively demonstrating that the refrangibility of a ray is linked to its colour, hence arguing that colour is an intrinsic property of light and does not arise from passing through a medium. Newton's later significance as a world-famous scientific genius and the apparent confirmation of his experimental results have tended to obscure the realities of his reception at the time. This paper explores the rhetorical strategies Newton deployed to convince his audience that his conclusions were certain and unchallengeable. This commentary was written to celebrate the 350th anniversary of the journal *Philosophical Transactions of the Royal Society*.

That God is colouring Newton does shew, And the devil is a Black outline, all of us know.

    William Blake, ‘To Venetian Artists’^1^

## 1. Introduction

Isaac Newton's outstanding reputation strongly affects how he is appraised retrospectively. Because he has become an international icon of scientific genius, it can be hard to appreciate that he was scarcely known outside Cambridge before his ground-breaking paper on optics was published in 1672 [1]. For over a quarter of a millennium, generations of scholars have sifted through the surviving evidence about the processes that enabled Newton to achieve his great discoveries, repeatedly producing new analyses of his intellectual sources, his experimental acumen and his character. Many historians of the Victorian era regarded Newton as a supremely rational scientist with impeccable morals, and they would have been horrified to read some recent accounts that focus on his alchemical research, biblical exegesis and sexual attitudes. Resistance to considering that a hero may be flawed—or humanly fallible—still colours modern views, although the same basic facts can often be interpreted in very different ways.

**Figure 1.**
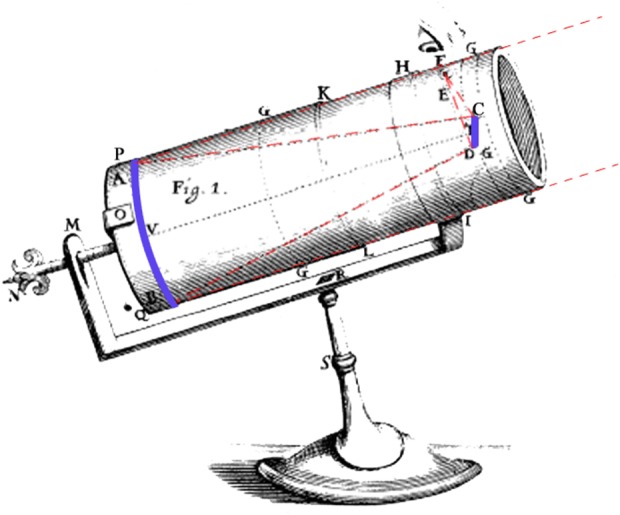
Newton's reflecting telescope.

Newton is reputed to have been a reclusive scholar who shunned controversy, but an alternative reading of his rise to fame suggests that on those occasions when he did choose to promote himself, he deployed skills worthy of a professional publicity agent [2]. To advertise a reflecting telescope he had built in Cambridge in 1669, he emphasized to his colleagues that it could magnify 150 times despite being only 6 inches long—and, he added, he had cast and ground the mirror himself from a home-made alloy (figure 1). It seems likely that Newton unobtrusively fanned rumours about his new invention, until within a couple of years his former mathematics tutor at Cambridge, Isaac Barrow, had arranged for it to be demonstrated to the Fellows of the Royal Society. The astonishingly small but powerful telescope caused a minor sensation. Having already succeeded Barrow as Lucasian professor, Newton was elected to a Fellowship on 11 January 1672 [3,4].

Determined to establish English priority, Henry Oldenburg, secretary of the Society and founder of the *Philosophical Transactions*, promptly wrote to Paris with the news. He also warmly congratulated Newton, who brushed aside Oldenburg's compliments with false modesty, but immediately replied with a carefully phrased suggestion. ‘I shall endeavour,’ he suggested in conventional self-effacing rhetoric, ‘to testify my gratitude by communicating what my poore & solitary endeavours can effect towards ye promoting your Philosophicall designes.’ [5].

Less than a month later, Newton despatched a letter that had little new to say about telescopes, but that overturned the prevailing theories of light, vision and colour and—after a small amount of editing—was reproduced in the *Philosophical Transactions* as ‘A Letter … Containing His New Theory about Light and Colors … ’ [1]. Written in a deceptively plain narrative style (‘having darkened my chamber, and made a small hole in my window-shuts … ’ [p. 3076]), this seminal article describes how Newton converted a prism, a child's plaything he had picked up at a local fair, into a sophisticated instrument. Glossing over many details, Newton outlined a crucial experiment to vindicate his conviction that sunlight contains light rays of differing colours and unequal refrangibility. He was concerned to demonstrate not only that he was right but also that two other people were wrong: René Descartes, his illustrious French predecessor, and Robert Hooke, his contemporary rival in England.

Whereas previous theories had taken white light for granted and tried to explain how colours are formed, Newton reversed the position by assuming that colour is a basic property that can be used to explain white light. In retrospect, Newton's experiments seem to expose natural reality in a delightfully clear and incontrovertible way, but they proved extremely hard for his readers to replicate. Achieving his results demanded a sophisticated grasp of glass technology as well as a delicate and patient experimental hand. In the interests of rhetorical persuasiveness, Newton did not describe the same sequence of processes that he used to reach his conclusions; omitting any mention of dead-ends, he left some trials unrecorded even in manuscript.

This review focuses on the 1672 paper and its impact rather than discussing the more complete version of Newton's theories presented in the various editions of his later book, *Opticks*.^2^ First, I sketch existing views of light (such puns are hard to avoid because of the close links between physically seeing and rationally knowing). I then summarize Newton's preliminary experiments before embarking on a closer reading of the paper itself. In the third and final section, I discuss his paper's immediate reception and its long-term influence.

## 2. Prequel: seventeenth-century optics

Experimental research into the anatomy of the eye and into the laws of optics both expanded rapidly in the early seventeenth century [6–8]. In 1604, the astronomer Johannes Kepler provided a new model of the eye by affirming that the seat of vision did not lie in the lens, as had been pronounced by the Graeco-Roman surgeon Galen almost 1500 years earlier. The eye, explained Kepler, resembles a *camera obscura* (a pin-hole camera), in which the lens plays a less crucial role, serving merely as a focusing device to form an inverted, reversed image of the outside world on the retina.

Although Descartes neglected to credit Kepler, he developed his ideas further in the 1630s. Now most famous as an abstract thinker, Descartes was also a practical man interested in improving instruments, and he visited his local butcher's shop to buy animals' eyes for his experiments. As Newton would do later, Descartes explored how physical impressions caused by light can be interpreted by the brain to yield visual information. In Descartes's dualist world, matter and spirit are entirely distinct. One of his illustrations shows a heavily bearded man staring at the back of a giant eyeball while dotted rays are focused on its retina, as though the brain belonging to a scientific observer could somehow have a detached, objective view of an image formed on its own body's internal projection screen. While retaining the concept of a human soul, Descartes never satisfactorily resolved the question of how mind and body can interact.

Descartes added to anatomical knowledge of the eye, yet his theories about vision remained underpinned by Aristotelian notions that had dominated European thought for almost half a millennium. Faced with the challenge of explaining colours, he reformulated mediaeval modification theories, arguing that colours arise during light's passage through the medium lying between an object and its observer. In his opinion, light consists of pulses, or changes of pressure, that are instantaneously transmitted through an all-pervasive but invisible aether of tiny spherical particles, which can be made to rotate. When a pulse of light brushes against stationary aether particles, the friction imparts spin: if they rotate faster than usual, they cause the sensation of redness, and if they spin slower, they give rise to blueness. Descartes derived the laws of refraction kinematically, starting from the counterintuitive assumption that light travels more quickly in a dense medium such as glass than in either water or air. By analysing how light is refracted through raindrops, he was able to demonstrate geometrically that primary and secondary rainbows would be perceived.

Philosophical and experimental interest in colour was stimulated by the phenomenon now known as Newton's rings, first investigated by Robert Hooke, experimental curator at the Royal Society. After exploring further some chance observations made with his microscope, Hooke discovered that concentric coloured rings could be created by thin plates of glass or films of liquid. Like Descartes, he explained light in terms of vibrations or pressure changes, but regarded colour as a distortion of the pulse rather than as a change in the medium. Rejecting both these views, Newton maintained that colours already exist (in some sense) in sunlight, but despite Hooke's earlier and continuing research, Newton always minimized Hooke's influence, claiming that his conclusions were his own. Enmity between the two men escalated as Hooke accused Newton of stealing his ideas not only on optics, but also on the mathematics of elliptical orbits. It is surely no coincidence that Newton published his second major book, *Opticks*, in 1704, the year after Hooke had died, even though he had been planning it since the 1680s.

Newton began to study light at least 7 years before his 1672 paper, while he was a student at Cambridge. He must have been forced to restrict his research to the summer months, when there was sufficient sunlight to produce useful results, but inconsistencies in his recollections make it impossible to pin down all the dates precisely. At first, he used very ordinary materials, placing a prism on a woven cloth to note that blue threads were displaced differently from red ones. Like other researchers of this period, he converted himself into an experimental subject, diligently recording his responses to different interventions. He almost blinded himself by staring at the sun with one eye to discover whether the physical effects that activity caused were different from ones he could summon up through his imagination, and also by pressing a blunt needle on the back of his eyeball to find out how his vision was affected.

In his early research, Newton attached great significance to the value of prism experiments for deriving quantitative explanations of optical phenomena. Gradually he developed his ideas, lecturing to students at Cambridge and building up the repertoire of experiments that he announced to the Royal Society in 1672. He insisted on what might nowadays seem obvious—that colour is an inherent property of light, not of the medium it travels through or is reflected from. Mainly because light travels in straight lines and does not curve round objects like sound, he refused to accept that it was a wave, although he failed to specify clearly what material form it might take.

Newton was interested in optics not only theoretically, but also practically, and he investigated how grinding lenses into different shapes could reduce the problems of spherical aberration. Some 3 years after he made his first telescope, he felt his ‘New Theory about Light and Colors’ was ready for public presentation. Ironically, his decision to build the reflecting telescope that first made him famous originated from his mistaken conviction that chromatic aberration in lenses could not be corrected.

## 3. Main feature: the 1672 paper

In 1672, the few scientific societies and journals that existed had only very recently been established, and they fulfilled different functions from those of today. In many ways, the Royal Society resembled a private club that provided light intellectual stimulation for leisured gentlemen. Travelling long distances had always been difficult and time-consuming, but by the mid-seventeenth century there was an effective correspondence network that stretched across Europe. Letters, natural history specimens and drawings regularly criss-crossed the continent, exchanged between members of an imagined international community known as the Republic of Letters. From its first issue in 1665, the *Philosophical Transactions* formalized these personal communications, making them available to readers both abroad and in England who lived too far away to attend meetings in London. Unlike a modern academic journal, the subject matter was not restricted to a specialized discipline. The items that appeared in the same issue as Newton's included a traveller's description of Eastern India and an essay on music.

At first glance, Newton's letter shares few obvious characteristics with modern scientific papers. There are only two small diagrams, footnotes had not yet been introduced, and—as if he were the only person who had ever studied light—Newton makes only one cursory reference [p. 3084] to any of his predecessors or colleagues. Even the year of publication looks strange: 1671/1672. Before 1752, England was out of kilter with most of Europe, because it followed the Julian rather than the Gregorian calendar. When 11 days were removed to bring it in line, the first day of each year was shifted from 25 March to 1 January.

Right from the beginning, the unknown young Newton casts himself in a superior position as the lynx-eyed detective, the expert on lenses who decided to amuse himself by playing with colours but who rapidly realized that major scientific issues were at stake. In his first sentence, in a deceptively casual remark inside parentheses, he points to a long-standing problem that Descartes had failed to resolve: why does a spherical lens not bring sunlight to a perfect focus? In a key strategic move, Newton switches to a different and little-studied instrument—the prism—for exploring the nature of light. Rewriting his repeated trials, mistakes and cul-de-sacs as a logical and systematic investigation, he makes his conclusions sound inevitable and hence unchallengeable—and by concealing vital details, such as the type of glass or shape of the prisms, Newton renders replication extremely difficult.

The paper has no sub-titles, but Newton presents his case in four stages, inviting his readers to follow him on his journey from plain experimental fact towards a quantitative theory of light supported by empirical verification. For more details, see Zemplén's ‘*The history of vision, colour & light theories*’ [9].

### (a) Stage 1

**Figure 2.**
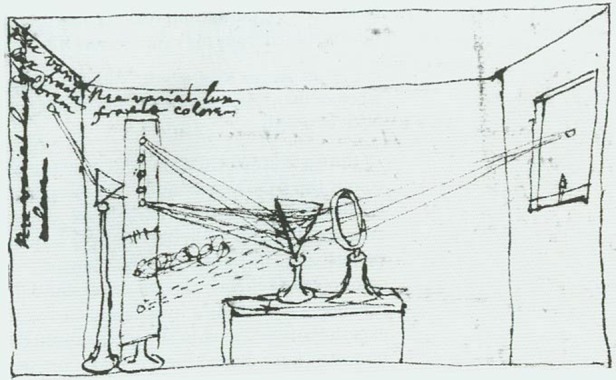
Newton's sketch of his crucial experiment.

In this artfully naive account, Newton reports that he started out by observing how a circular beam produces a rectangular spectrum on being passed through a prism (even though it is more pear-shaped). He then appears to examine and dismiss reasonable explanations, substantiating his arguments with quantitative calculations. What he is actually doing is rejecting Descartes' arguments that colours are modified by the medium: Newton leaves unstated the alternative possibility that the sine laws of refraction are at fault. By comparing rays with tennis balls (then small cork balls covered with woollen cloth), he implies that light consists of particles; and by giving precise data, he conceals the fact that whereas some of his figures are measured (how remains unspecified), others are calculated. Claiming to have eliminated all the arguments that would favour a medium-based explanation, he sets up what he calls a crucial experiment, although (as his critics soon pointed out) it is not at all clear which two hypotheses Newton is discriminating between. Ostensibly testing his suggestion that rays are unequally refracted—significantly, at this stage he does not mention colours—in actuality he is confirming it. Although no illustration is given in the article, figure 2 reproduces a sketch made by Newton showing how he converted his study into a large *camera obscura* by letting a small ray of sunlight pass through a hole in the shutters. His innovation is to use two prisms sequentially. By boring a hole in a screen between them, he singles out individual sections of the first prism's spectrum; these then pass through the second prism to arrive at different places on a second screen. From his demonstration of differential refraction, Newton draws a major theoretical inference: light is, he writes (his italics), ‘a *Heterogeneous mixture of differently refrangible rays*’ (p. 3079).

### (b) Stage 2

As though remembering that he had promised Oldenburg a letter on telescopes, Newton then devotes a few paragraphs to the implications of his research for optical instruments, and his motivation for constructing the reflecting telescope he had sent in earlier. Extrapolating from prisms, he is now able to explain spherical aberration. Stressing (wrongly) the possibility of eliminating chromatic aberration (my term, not his), he boasts of overcoming initial setbacks through his skills at making parabolic mirrors, and sketches a possible reflecting microscope. Although reflecting telescopes do indeed have great advantages, unsurprisingly he does not mention three drawbacks: his highly polished mirrors corroded after a few weeks; in large telescopes, the mirror's own weight deforms it; and chromatic aberration cannot be entirely eliminated because catoptric instruments use lenses in the eye-pieces.

### (c) Stage 3

With apparent relief, Newton ‘return[s] from this digression’ (p. 3081) to lay out a theory of light in 13 numbered propositions, a Euclidean style of presentation he later adopted for the *Principia*. Abruptly abandoning his persona of candid Baconian reporter, he announces that it would be too tedious to describe all his experiments: instead, he will lay down a doctrine and then illustrate how it can be explored experimentally. To mathematize his theory of colours, Newton has to show that the refrangibility of a ray is intrinsically linked to its colour, a task he undertakes in the first three propositions, in which he rejects modification theories by insisting that colours are a primary rather than a secondary quality, and so unaffected by reflection or refraction. The next six propositions are taken up with examining how colours can be combined and separated, with particular emphasis on whiteness, ‘the most surprising and wonderful composition’ (p. 3083) that exists not on its own but only by recombining individual coloured rays. At this point in his life, Newton perceives five basic spectral colours: it was only later that he included indigo and orange so that rainbows would conform to his Pythagorean vision of a harmonious universe whose mathematical characteristics corresponded with the seven notes of the musical scale. To demonstrate the value of his new theory, Newton devotes his final four propositions to considering briefly how coloured phenomena are perceived. As well as analysing rainbows, he discusses why objects are seen with different colours under particular circumstances, attributing the reason not to any intrinsic colour of any particular material but instead to the way in which it reflects or refracts light.

### (d) Stage 4

To conclude, Newton pursues a more philosophical vein. Colours, he insists, lie not in the medium and not in objects but in light rays—and since colours are qualities, the rays themselves must be made of a material substance. Claiming that his account would undoubtedly stimulate further research, Newton thoughtfully provides full instructions, including dimensions, for an experimental set-up that he had designed to demonstrate—not test—the validity of his propositions. He recommends passing sunlight first through a prism and then through a lens on to a sheet of paper. By moving the paper backwards and forwards through the focal point, the refracted beam can, he reports, be seen splitting into colours and then reuniting into white light. However, even the incomplete trouble-shooting details he provides confirm how tricky it can be to obtain the desired effects. Adopting a tone suggesting he expects no response, Newton ends by inviting readers to let him know if he has made any mistakes.

The very last paragraph, presumably added by Oldenburg, warmly commends Newton's letter to its readers, but omits to mention that it had been surreptitiously edited. The original has been lost, but in a copy annotated by Newton, the following sentences appeared immediately after ‘wherein the *Origin of Colours is unfolded*’ (p. 3081), and just before he switched out of his Baconian, historical style of narrative:

A naturalist would scarce expect to see ye science of those become mathematicall, & yet I dare affirm that there is as much certainty in it as in any other part of Opticks. For what I shall tell concerning them is not an Hypothesis but most rigid consequence, not conjectured by barely inferring 'tis thus because not otherwise or because it satisfies all phænomena (the Philosophers universall Topick,) but evinced by ye mediation of experiments concluding directly & without any suspicion of doubt. [10]

The suppression of these controversial claims is significant. Newton argues here not only that colour can be quantified but also that differential refrangibility is an established fact, not a testable hypothesis—yet this assertion rests on the unstated assumption that the geometrical laws of optics are valid. Whoever removed these two sentences recognized the strength of its epistemic claims in the context of an article lacking experimental detail.

## 4. Sequel: consequences of Newton's 1672 paper

Newton's 1672 paper carried two main implications for the future: the nature of light and the properties of colour. Most obviously, Newton himself continued to experiment and to revise these early ideas. In addition, aiming to fulfil their own various agendas, natural philosophers picked up, developed and consolidated hints and suggestions made by Newton at different times. As a consequence, what became known as Newtonian views were not necessarily identical either to each other or to the opinions voiced by Newton at any particular time.

By the middle of the eighteenth century, Newton's prestige as the hero of an enlightened nation was secure. The 1755 marble statue in the ante-chapel of Trinity College Cambridge presents an elegantly dressed Newton who is not controlling the planets, but wielding a prism as if it were an orator's baton (his apple was a nineteenth-century innovation). To see was to know, and by then even French natural philosophers recognized Newton's supremacy as the natural philosopher who had illuminated the dark clouds of superstition with the light of reason.

Establishing this authority had entailed persistent hard work and subtle manoeuvres on the part of Newton and his allies. Ideologically, an intellectual Republic of Letters transcended national boundaries, but, in reality, debates about optics or gravity were inseparable from political, religious and commercial differences. What might appear to be abstract discussions of refractive indices or the meaning of infinity were shot through with local interests, including those of the Venetian glass trade, the Hanoverian court and the Jesuit order.

Within a week, Hooke fired off his first attack on Newton's report, and other critics—both at home and abroad—rapidly followed suit. They were dissatisfied not only with the difficulties of replicating his results, but also with his methodological arguments, and a couple of months later Newton wrote to Oldenburg admitting that he should have given fuller experimental details. The status of the experiment that he claimed to be crucial continued to be challenged. Should that one single trial override the implications of many others? When competitors announced they could split Newton's supposedly basic rays into ones of different colours, he retorted that if his rivals could not replicate his work, they were using the wrong type of prism. But how do you define the right sort of prism? Newton seemed to be adopting the unsatisfactory criterion that the right sort of prism was the one that demonstrated he was right [11].

Prisms now seem unproblematic displayers of nature's truths, but at the time, the status of the prism as a philosophical instrument was contested. Although Hooke managed to confirm some of Newton's findings, Newton's opponents in France produced conflicting experimental results and rival theoretical explanations. Over 30 years later, Newton tried to quell the protracted debates by publishing *Opticks* (1704), intended partly as a manifesto for British empiricism, which he promoted as a far more reliable route to truth than French hypothesizing. Producing reconstructed versions of his earlier experiments, he at last provided full specifications of his experimental set-ups and of the prism glass. Until his death in 1727, Newton continued to strengthen his influence through behind-the-scenes negotiation, carefully instructing his protégé John Desaguliers how best to overcome French opposition. Desaguliers expressed this cross-Channel antagonism colourfully: ‘It is to Isaac Newton's Application of Geometry to Philosophy, that we owe the routing of this Army of Goths and Vandals [Cartesians] in the Philosophical World.’ [12]

Newton continued to maintain that light is a material substance, producing various hypotheses and trying to incorporate wave-like features. Rather than develop those suggestions, during the first half of the eighteenth century his supporters began to make more definitive pronouncements about light's corpuscular nature than he had made in his original article. This was a period when scientific lecturers and writers—men like Desaguliers—were starting to forge a career as public educators, and they needed a straightforward message to deliver. By visualizing light as a beam of rapidly moving tiny particles, they could provide satisfactory explanations of phenomena such as reflection and refraction. At the same time, a substantial minority of anti-Newtonians, now largely forgotten, insisted that light was some type of divine fluid. For them, the metaphorical resonances of biblical imagery outweighed any materialist interpretation of light as particles [13].

From mid-century onwards, various wave and vibration theories became increasingly popular amongst pro-Newtonian researchers, who started examining the projectile theory critically rather than simply accepting and propagating it. Newton himself had suggested that the whole universe might be filled with a weightless invisible aether comprising tiny undetectable particles, and this model came to represent orthodox Newtonianism. Despite the demands an aether might seem to place on imagination and credulity, for many people it was preferable to believing that ordinary inert matter could somehow exert a gravitational power stretching out many thousands of miles through empty space: that sounded too much like magical occult forces, and gave to matter a power conventionally reserved for God. This shift was given additional impetus in 1758, when a London optician demonstrated that even the great Newton could be wrong by inventing an achromatic lens. Now that their hero had been proved fallible, even critics who described themselves as Newtonians felt less inhibited about modifying his ideas.

The credit for confirming the existence of light waves analogous to those of sound is often given to Thomas Young, a polymathic doctor and lecturer at the newly founded Royal Institution in the early nineteenth century. According to condensed versions of the past, Young performed a crucial experiment by passing a beam of light through two tiny slits in a screen and observing an interference pattern of alternating dark and white stripes, thus showing that light is not particulate but ripples out from a source like waves from stones dropped into a pond. In reality, there was no single specific eureka moment of discovery. Young examined many optical effects, including the rings first studied by Hooke; furthermore, the modern wave theory of optics owes much to the contributions of French experimenters. Young and his compatriots were unfamiliar with the analytical calculus that had been developed on the continent but was frowned upon in Britain. In the Laplacian research group based just outside Paris, Augustin Fresnel and his colleagues developed important mathematical models to describe the behaviour of light.

In this period before the word ‘scientist’ was invented (by William Whewell in 1833), there were no clear distinctions between philosophers, experimenters or artists. Newton is now celebrated as a great physicist, but Newton's impact spread across many areas that now seem distinct, and his ideas were discussed as eagerly by artists as by the Fellows of the Royal Society. For example, the landscape painter John Constable sketched ray diagrams of reflection and refraction in a rain drop, declaring in a lecture at the Royal Institution that his art was a science. Conversely, the significance of Newton's research for the practical problems of mixing colours was first expounded in a book on perspective by the mathematician Brook Taylor.

Constable also pointed to an inherent shortcoming of Newton's analysis: the impossibility of stating how many colours there are. Rainbows with clear stripes appear only in books; in actuality, the hues blend imperceptibly from one to the next as if there were an infinite number of coloured rays. In 1672, the same year that Newton presented his paper to the Royal Society, a leading spokesman at Paris's Royal Academy emphasized that a viable theory of colour was needed in order to rescue art from its contemporary domination by monochromatic drawings. Initially, Newton introduced confusion by suggesting that there are five fundamental or uncompounded colours, and that white light can be reconstituted by combining them together. His critics promptly claimed that white light can be formed by bringing together just the outer two colours of the spectrum (perhaps their definition of ‘whiteness’ was somewhat liberal). They also pointed out that Newton identified green both as a basic colour and as a blue–yellow mixture, and that to say there are five primary colours is to make an arbitrary choice. ‘3 primaries, 7 prismatics,’ noted Constable tersely, but ‘there is no limit to the number of prismatic colours.’ [14,15].

**Figure 3.**
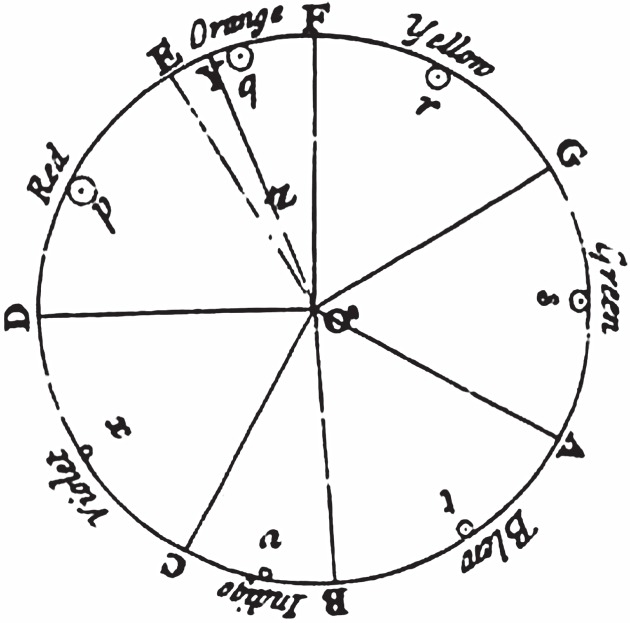
Newton's colour circle published in *Opticks* (1704).

Searching for a principle of harmony, Newton sought to analyse colours mathematically. By analogy with the musical octave, he developed a colour circle divided into seven unequal segments (figure 3). The arc length of each segment is proportional to its position in the scale, and the radial position (*Z*) of a mixed colour indicates how it has been formed. However, Newton never specified exactly what those quantities might represent in terms of light and pigments. Well into the nineteenth century, Newton's colour wheel was elaborated but also contradicted by several authors, who designed a range of complicated circular and pyramidal diagrams.

In nineteenth-century Britain, Newton enjoyed such prestige that adopting the wave theory failed to dent his reputation. It became impossible to oppose Newton outright: any revisions or corrections to his ideas had to be incorporated within a Newtonian framework. For instance, David Brewster was one of Newton's most fervent admirers at the Royal Society, yet he substantially modified Newton's colour theory after examining the prismatic spectrum through glasses of different tints, and announced that there are only three true primary colours: red, yellow and blue. Enormously influential on artists such as J M W Turner, Brewster became notorious for his habit of visiting exhibitions to inspect paintings with his pocket set of coloured glasses before angrily denouncing any errors he perceived.

Europeans were less constrained by patriotism. The most overtly hostile objections came from Johann Wolfgang von Goethe, who flatly rejected Newton's claim that white light is a mixture of coloured rays. Instead, insisted Goethe, colour arises from mixing white light with darkness, its polar opposite, and he showed how coloured fringes can appear at the junction of white and black edges. He also rejected notions of scientific objectivity by incorporating the physiological and emotional responses of an individual observer into the experimental process. Whereas Newton projected a spectrum on to a wall so that several people could view it simultaneously, Goethe made his own retina the screen by holding the prism directly in front of his eye to experience colours radiating out in different directions. Although dismissed by British scientists, Goethe's ideas were very influential on German science and art during the nineteenth century.

Optical theories were continually revised in the centuries following Newton, but the most fundamental shift was introduced by Albert Einstein, who suggested in 1905 that light waves are made up from quanta of energy. Although this novel concept was not accepted for several years, some scientists maintained that photons seemed to vindicate Newton's vision of particulate light rays. However, not only had he prevaricated on that point, he also had no theoretical concept of energy, which was not established in physics until the nineteenth century. Even so, Einstein linked himself to the predecessor he admired so much by providing an adulatory preface for a new edition of *Opticks*. ‘Fortunate Newton, happy childhood of science!’ he enthused; ‘[Newton's theories] seemed to flow spontaneously from experience itself, from the beautiful experiments which he ranged in order like playthings and describes with an affectionate wealth of detail.’ [16].

Writing over a quarter of a millennium after Newton's first paper, Einstein did not point out that in 1672, not even Newton's patrons would have supported this endorsement. As perceived by his contemporaries, Newton was a brash yet talented newcomer. His radical new theory of light was exciting—but not fully warranted by the experimental descriptions he provided. They would have been astounded to learn that he is now internationally renowned as a scientific genius, a category unknown before the Romantic period.

## Footnotes

1 Blake W. 1972 *Complete writings*, p. 554. Oxford, UK: Oxford University Press.

2 For more information see the major work devoted to Newton's optics by A. R. Hall [4].

## Author profile

**Patricia Fara** has an Oxford degree in Physics, a London PhD in History of Science, and now lectures in the History and Philosophy of Science department at Cambridge, where she is the Senior Tutor of Clare College. Her first academic monograph was *Sympathetic Attractions: Magnetic Practices, Beliefs, and Symbolism in Eighteenth-Century England* (1996), which inevitably discussed Isaac Newton. She later focused on Newton's shifting reputation, and produced her first popular book, *Newton: The Making of Genius* (2002). Since then, she has published widely, including *An Entertainment for Angels: Electricity in the Enlightenment* (2002) and *Pandora's Breeches: Women, Science and Power in the Enlightenment* (2004). More recently, her prize-winning *Science*: *A Four Thousand Year History* (2009) has been translated into nine languages. Following *Erasmus Darwin: Sex, Science and Serendipity* (2012), she is currently writing a book on women, science and suffrage in the First World War. In addition to her academic work, she is a regular contributor to popular journals as well as TV and radio programmes (such as *In our Time*).

## References

[1] Newton I 1972 A Letter of Mr. Isaac Newton, Professor of the Mathematicks in the University of Cambridge; Containing His New Theory about Light and Colors: Sent by the Author to the Publisher from Cambridge, Febr. 6. 1671/72; In Order to be Communicated to the R. Society. Phil. Trans. 6, 3075–3087. (10.1098/rstl.1671.0072)

[2] Fara P 2002 Newton: the making of genius. London, UK: Macmillan.

[3] Westfall R 1980 Never at rest: a biography of Isaac Newton. Cambridge, UK: Cambridge University Press.

[4] Hall AR 1993 All was light: an introduction to Newton's Opticks. Oxford, UK: Clarendon Press.

[5] Turnbull H 1959–1977 The correspondence of Isaac Newton, vol. 7, p. 81 Cambridge, UK: Cambridge University Press.

[6] Park D 1997 The fire within the eye: a historical essay on the nature and meaning of light. Princeton, NJ: Princeton University Press.

[7] Wade NJ 1998 A natural history of vision. Cambridge, MA: MIT Press.

[8] Darrigol O 2012 A history of optics: from Greek antiquity to the nineteenth century. Oxford, UK: Oxford University Press.

[9] Zemplén G 2005 The history of vision, colour & light theories: introduction, text, problems, pp. 263–289. Bern, Switzerland: Bern University.

[10] Turnbull H 1959–1977 The correspondence of Isaac Newton, (note 3), vol. 1, pp. 96–97. Cambridge, UK: Cambridge University Press.

[11] Schaffer S 1989 Glass works: Newton's prisms and the uses of experiment. In The uses of experiment: studies in the natural sciences (eds Gooding D Pinch T Schaffer S), pp. 67–104. Cambridge, UK: Cambridge University Press.

[12] Desaguliers J 1734 A course of experimental philosophy, p. vi London, UK.

[13] Cantor G 1983 Optics after Newton: theories of light in Britain and Ireland, 1704–1840, pp. 25–146. Manchester, UK: Manchester University Press.

[14] Gage J 1999 Colour and meaning: art, science and symbolism, pp. 134–152. London, UK: Thames & Hudson.

[15] Kemp M 1990 The science of art: optical themes in western art from Brunelleschi to Seurat, pp. 285–322. New Haven, CT: Yale University Press.

[16] Einstein A 1952 ‘Foreword’ in Opticks, 1952 Edition. New York, NY: Dover.

